# Supplementary material for: Unraveling the role of γδ T cells in the pathogenesis of an oncogenic avian herpesvirus
Source: mBio. 2024 Jul 2;15(8):e00315-24. doi: 10.1128/mbio.00315-24 (PMC11323538; doi:10.1128/mbio.00315-24)
Supplement: Fig. S1 — Flow cytometry gating. [file mbio.00315-24-s0001.pdf]

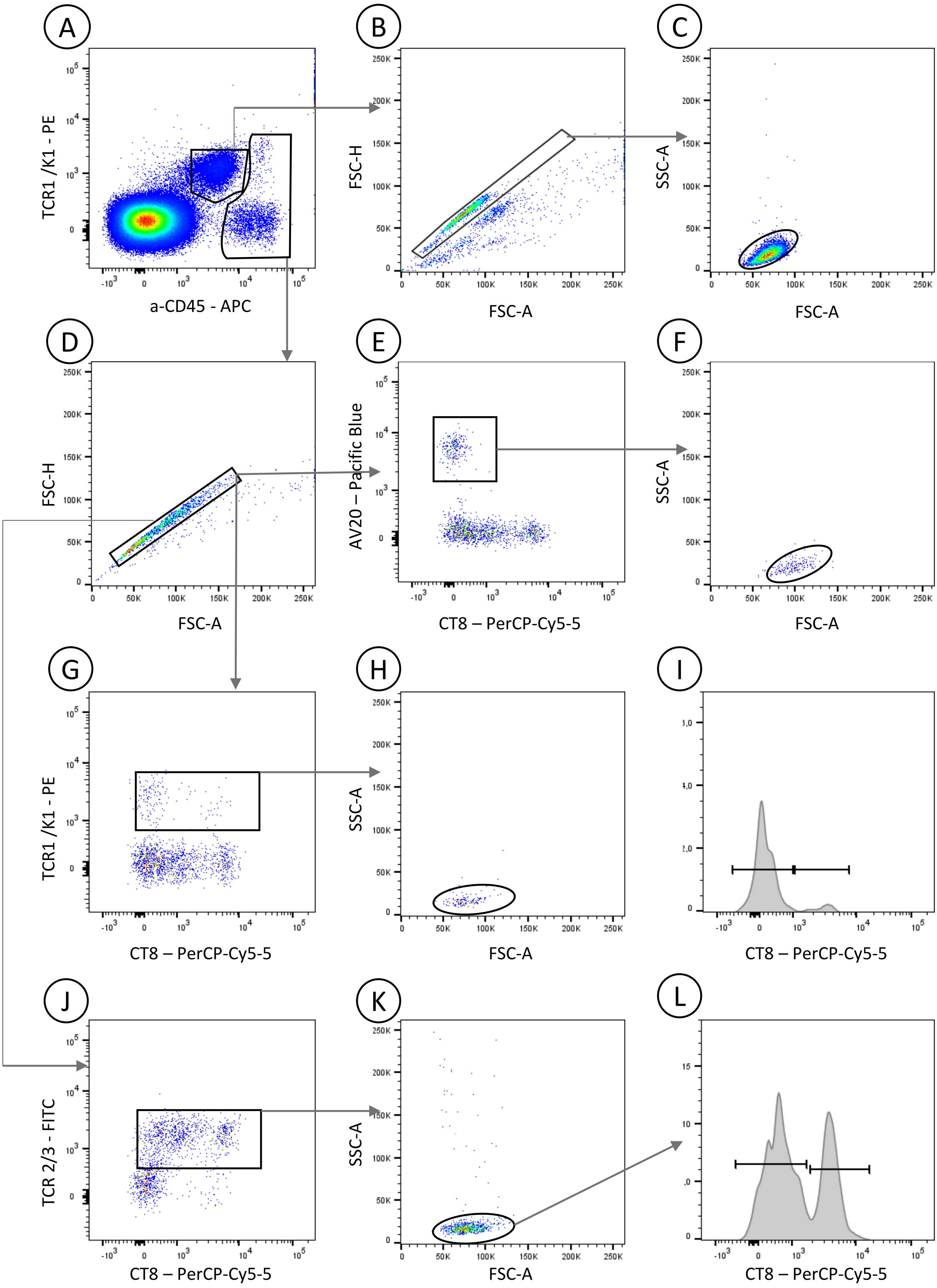

Figure Flow cytometry gating

EDTA-blood was stained with mix of anti-TCRαβ/Vβ1-FITC), anti-TCRαβ/Vβ2-FITC (clone TCR3), anti-TCRγδ-PE (clone TCR1), anti-Bu1-Pacific Blue (clone AV20), anti-CD8-PerCP-Cy5.5 (clone CT8, anti-CD45-APC (clone UM16-6,) and thrombocyte marker in a no-lyse no-wash one-tube procedure and subsequently analyzed by flow cytometry.

We first separated thrombocytes and leukocytes (A) followed by a single cell gate (B and D). Leukocytes were subdivided in B cells (E), yd-T cells (G) and aß-T cells (J). T cell subpopulations were further separated in CD8pos and CD8neg cells (I and L). CD8neg aß-T cells were addressed as CD4pos T cells. To exclude potentially contaminating erythrocytes, for all cell populations an additional FSC/SSC gating was performed (C, F, H and K).
